# Plastic Crushing Failure of Bio-Inspired Cellular Hierarchical Topological Sandwich Core

**DOI:** 10.3390/ma14175040

**Published:** 2021-09-03

**Authors:** Yuwu Zhang, Yuliang Lin, Xiangcheng Li

**Affiliations:** College of Liberal Arts and Sciences, National University of Defence Technology, Changsha 410073, China; zhangyuwu12@nudt.edu.cn (Y.Z.); lixiangcheng7558@nudt.edu.cn (X.L.)

**Keywords:** structural hierarchy, in-plane response, deformation mode, failure mechanism, constituent material effect

## Abstract

Bio-inspired self-similar hierarchical honeycombs are multifunctional cellular topologies used for resisting various loadings. However, the crushing behavior under large plastic deformation is still unknown. This paper investigates the in-plane compressive response of selective laser melting (SLM) fabricated hierarchical honeycombs. The effects of hierarchical order, relative density as well as constituent material are evaluated. The results show that at small deformation, the AlSi10Mg alloy hierarchical honeycombs show great advantages over the elastic modulus and compressive strength than 316L steel hierarchical honeycombs. As the relative density and hierarchical order increase, the failure mechanism of AlSi10Mg alloy honeycombs gradually changes from a bending-dominated mode to a fracture-dominated mode; whereas all the 316L steel honeycombs fail due to the distortion of original unit cells. At large deformation, the AlSi10Mg alloy honeycombs behave with brittle responses, while the 316L steel honeycombs exhibit ductile responses, showing a negative Poisson’s ratio behavior and gradient deformation of hierarchical unit cells. The addition of unit cell refinements improves the elastic modulus of AlSi10Mg alloy honeycombs and advances the densification of 316L steel honeycombs. In addition, the effect of constituent material on the compressive response of hierarchical honeycombs has been discussed. This study facilitates the development and future potential application of multifunctional ultra-light sandwich structures.

## 1. Introduction

A vast majority of man-made engineering items are inspired by prototypes in nature. A most typical example of this is the periodic honeycombs which are motivated by macro honeybee combs. Honeycomb topologies have been used extensively as the core of lightweight sandwich structures, owing to their excellent engineering properties in terms of mechanical strength, thermal and sound insulation, energy absorption as well as vibration dampening [1,2,3,4,5]. Wadley [6] reported that the honeycomb structure exhibits the highest out-of-plane strength and elastic modulus among the competitive cellular topologies, followed in succession by corrugated and lattice truss structures. However, the mechanical behavior and energy absorption capacity of honeycomb is inferior along with the in-plane orientation, which needs to be enhanced for meeting specific engineering requirements. Wang and McDowell [7] conducted a systematic analysis on the in-plane compressive response between periodic honeycombs with different unit cell types and indicated that there exist two main domination modes for the in-plane deformation depending on the honeycomb type. Deformation of hexagonal [8] and square honeycombs [9] is dominated by bending; whereas deformation of triangular [10], diamond honeycomb [11] and Kagome honeycombs [12] is dominated by stretching. Among the bending dominated configurations, the application of hexagonal honeycomb attracts increasing attention recently, of which the in-plane strength and stiffness are proportional to the square and cube of the relative density of honeycombs, respectively [13,14].

**Figure 1:**
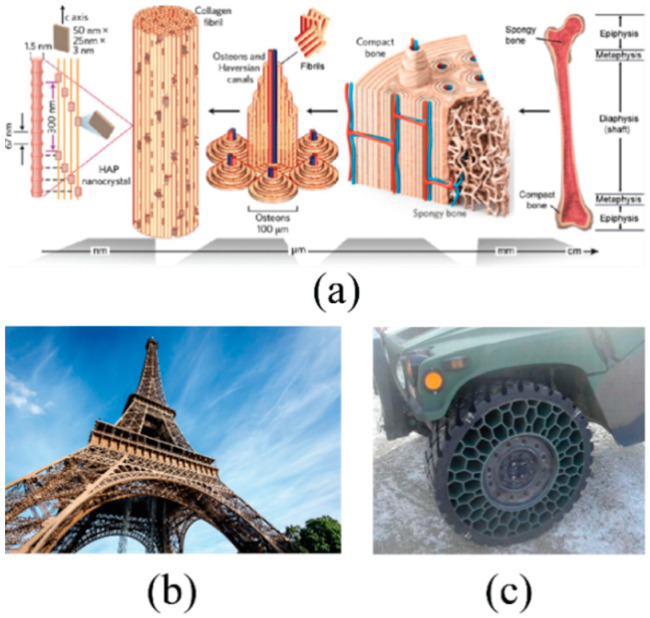
Examples of hierarchical honeycombs in practice: (**a**) microscopic hierarchical structures in biological bone and wood [15]; (**b**) the hierarchical structural Eiffel Tower; (**c**) novel automotive tire.

Motivated by the microstructure in bone and wood in nature [15], as shown in Figure 1a, bio-inspired self-similar hierarchical honeycomb with more advanced topology than conventional honeycomb has recently emerged via replacing the three-edged node joint with a smaller hexagonal unit cell, and higher hierarchical orders can be obtained by means of iteration [16]. Comparing with the conventional honeycombs, better in-plane mechanical properties have been observed for hierarchical honeycombs due to the novel weight-saving topology [17]. Oftadeh et al. [18] indicated that the elastic moduli of the first and second-order hierarchical honeycombs are 2 and 3.5 times higher than those of the conventional ones with identical relative densities. Larger in-plane compressive strengths are also observed when a set of specific geometrical parameters is applied [19,20]. In practice, hierarchical honeycombs have many applications. Two examples are given in Figure 1b,c.

It is known that the in-plane honeycomb topologies enable dissipating energy via plastic deformation of parent material at weak bands of cell walls. Adding a hierarchy increases the number of wall joints, contributing to a more uniform stress distribution than traditional honeycombs [21]. In addition, as a sandwich core, a hierarchical honeycomb extends the path of wave propagation and enables a reduction in potentially harmful momentum transfer [22,23]. Hence, hierarchical honeycombs are also promising for using in energy-absorption and buffering devices, with the in-plane compressive behavior being crucial.

However, most of the existing studies focus on the elastic properties of hierarchical honeycombs via theoretical or numerical methods, and its plastic response under a high deformation condition was rarely considered, since the additional hierarchical refinement poses a great challenge to analysis. Thus, understanding the effect of different hierarchical orders and the constituent material on the mechanical responses as well as energy absorption capacity is of great importance to advance the application of hierarchical honeycombs in more engineering fields.

The present study had two purposes: (i) to evaluate the geometrical effect and constituent material effect in determining the mechanical response of additively manufactured hierarchical honeycombs under large deformation; (ii) to verify experimentally the applicability of the existing theoretical models that are used for predicting the mechanical properties of hierarchical honeycombs.

## 2. Experimental Details

### 2.1. Hierarchical Honeycomb Topologies

**Figure 2:**
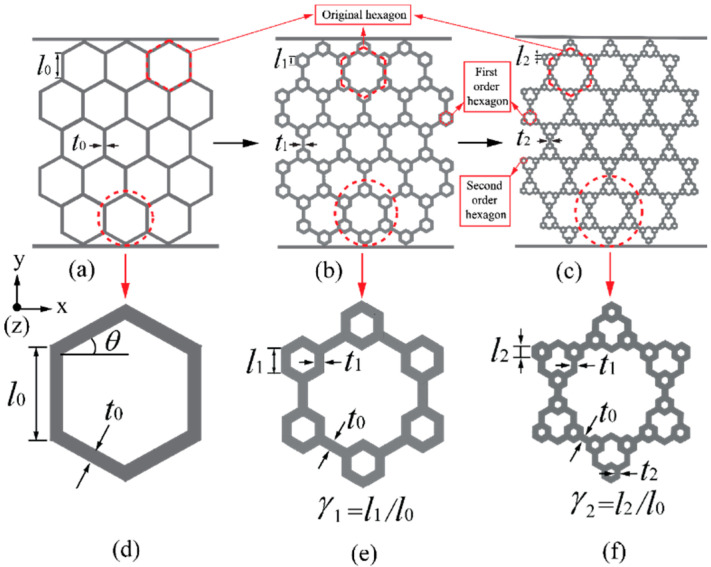
In-plane geometrical schematics of three types of honeycombs: (**a**) normal order; (**b**) first-order; (**c**) second-order; (**d**–**f**) are the unit cell geometries for (**a**–**c**), respectively.

In the present work, selective laser melting (SLM) tests were conducted on three-dimensional periodic honeycombs prepared in normal, first and second-orders. The schematics of in-plane topology as well as the corresponding hierarchical unit cells are illustrated in Figure 2a–f, respectively. In this paper, coordinates x and y are considered to be in-plane, and the coordinate z indicates an out-plane orientation (Figure 2). All the honeycombs contain a group of original basic hexagonal unit cells. The length of the edge is written as $l_{0}$ and the wall thickness is written as $t_{0}$. Moreover, the first and second-order honeycombs with in-plane hierarchical refinements are designed based on the combination of unit cells at two and three length scales, respectively. Regarding the first-order honeycomb, each vertex of the original unit cell is replaced by six secondary unit cells, of which the edge length and wall thickness are marked as $l_{1}$ and $t_{1}$, respectively. The second-order honeycomb is refined from the first-order honeycomb by further replacing the vertex of each first-order unit cell with cells with reduced dimension in edge length and wall thickness, which are recorded as $l_{2}$ and $t_{2}$, respectively. In this way, each normal order honeycomb specimen contains 17 original unit cells, each first-order honeycomb specimen contains an additional 50 first-order unit cells, and each second-order honeycomb specimen contains an additional 152 second-order unit cells. For each honeycomb with a hierarchical order of *i,* the in-plane configuration can be described by two geometrical parameters $\gamma_{i}$ and $\eta_{i}$, indicating the ratios of edge length and cell wall thickness, where $\gamma_{i}$ can be calculated by dividing $l_{i}$ (i.e., edge length of the secondary unit cell) by $l_{0}$ (i.e., edge length of the original unit cell) and $\eta_{i}$ can be calculated by taking the quotient of $t_{i}$ (i.e., wall thickness of the secondary unit cell) and $t_{0}$ (i.e., wall thickness of the original unit cell). For each type of honeycomb, there exists a constraint between $t_{i}$ and $l_{i}$, which can be written as $0\leq t_{i}\leq \sqrt{3}l_{i}/2$. When $\gamma_{1}=0$, a normal order honeycomb is constructed. For a first-order hierarchical honeycomb, $0\leq \gamma_{1}\leq 0.5$. For a second-order hierarchical honeycomb, $0\leq \gamma_{2}\leq \gamma_{1}$ exists when $\gamma_{1}\leq 0.25$, whereas $0\leq \gamma_{2}\leq \left(0.5-\gamma_{1}\right)$ when $0.25\leq \gamma_{1}\leq 0.5$. The relative density $\bar{\rho }$ can therefore be given as follow assuming the wall thicknesses of unit cells with distinct length scales are uniform [24]

(1) $$\bar{\rho }=\frac{\rho_{H}}{\rho_{s}}=\frac{2}{\sqrt{3}}\left(1+2\sum_{1}^{n}3^{i-1}\gamma_{i}\right)\frac{t}{l_{0}}$$

where $\rho_{H}$ is the density of the honeycomb; $\rho_{s}$ is density of the parent material; n represents the hierarchical order.

The specimens were prepared by AlSi10Mg alloy and 316L steel for exploring the effect of constituent material on their mechanical properties. To identify the influence of relative density, honeycombs with three distinct relative densities ($\bar{\rho }$ = 0.07, 0.11, 0.21) were prepared. Dimensions of the specimen are 46.4 mm in width (*W*) in x-axis and 53.6 mm in length (*L*) in y-axis. The specimen thickness (*H*) in the out-of-plane orientation (z) is 25 mm. For all the specimens, $l_{0}$ is designed to be 6.7 mm. $\gamma_{1}$,$\gamma_{2}$ are selected to be equal to 0.3 and 0.12. According to Equation (1), the uniform wall thickness can be calculated. The in-plane geometrical parameters of all the specimens are summarized in Table 1. As to the numbering of honeycombs in this table, AL and ST mean AlSi10Mg alloy and 316L steel, respectively; N, F and S indicate the normal, first and second-order. The number after them correspond to the $\bar{\rho }$. It should be noted that the relative densities of honeycomb specimens are not perfectly identical to the designed density due to the accuracy of the SLM. The measuring errors could be related to the particle size of the metallic powder or the changing layer thickness caused by additional powder melting.

### 2.2. Selective Laser Melting (SLM) Fabricating Method

**Figure 3:**
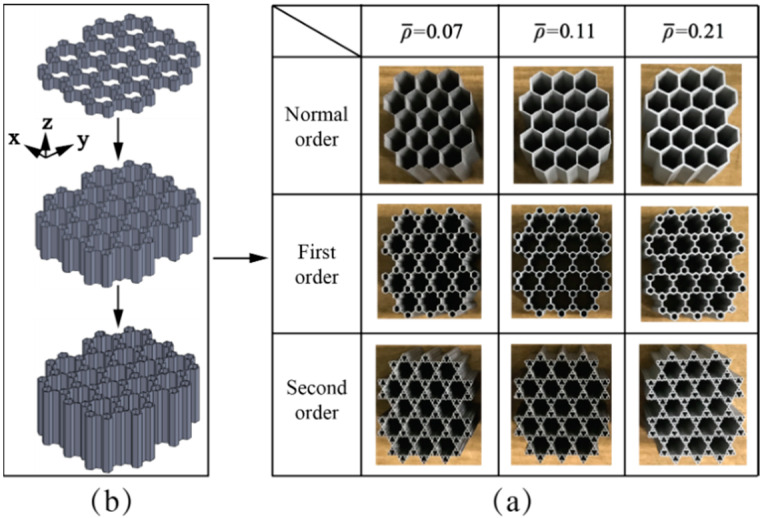
(**a**) The fabricating orientation diagram of honeycomb specimens, and (**b**) photograph of selective laser melting (SLM) fabricated normal order, first-order and second-order honeycomb specimens with three relative densities of 0.07, 0.11 and 0.21.

The hierarchical honeycomb specimens made by AlSi10Mg alloy and 316L steel were obtained via SLM manufacturing methodology. The SLM machine, which has a maximum fabricating dimension of 275 × 275 × 355 mm^3^ and a high-precision laser scanning galvanometer, was produced by the Farsoon Technologies company, Changsha, Hunan Province of China. For AlSi10Mg alloy specimens, the spherical diameter of the parent material powder ranges from 15 to 53 μm. The apparent density is 1.45 g/cm^3^. It is constituted by 88.9 wt% Al, 10.2 wt% Si, 0.5 wt% Mg and 0.03 wt% other metallic components, such as Mn, Ti, Cu, Ni. The SLM machine for AlSi10Mg alloy is designed with an optical fibre laser. The manufacturing method and the parameter selections are described below: the chessboard strategy with a laser scan speed of 1300 mm/s was applied; the deposited layer thickness was 35 μm for obtaining reliable mechanical properties; the temperature of the build platform was stabilized at 180 °C; the fabricating environment was mainly filled by nitrogen and oxygen, in which the oxygen accounts for no more than 0.1%. For 316L steel specimens, the parent material powder’s spherical diameter ranges from 15 to 53 μm with an apparent density of 3.9 g/cm^3^; the parent material powder is composed of 65.1 wt% Fe, 16.8 wt% Cr, 12.2 wt% Ni, 2.5 wt% Mo, 2.0 wt% Mn, 0.9 wt% Si and some other metallic components such as C, P, H and S. The SLM machine for AlSi10Mg alloy also come with an optical fibre laser. The manufacturing method and the selection of the parameters are described as follows: following a chessboard strategy, a scan speed of 1500 mm/s was used for laser scan; the layer thickness was controlled at 40 μm; nitrogen with less than 0.05% oxygen was selected as the fabricating atmosphere. All the honeycomb specimens were prepared with deposited layers in the z direction (or height direction) only for an in-plane compression. Figure 3 illustrates the fabricating orientation of honeycomb specimens, and the photographs of the normal order honeycomb specimens and hierarchical honeycomb specimens.

Uniaxial tensile tests were performed on dog-bone specimens to investigate the mechanical properties of the parent material. Stacking layouts along and perpendicular to the loading direction were designed for the specimens, so that the property anisotropy of SLM manufactured material can be explored. 

### 2.3. Mechanical Testing

#### 2.3.1. Uniaxial Tensile Test of Constituent Materials

**Figure 4:**
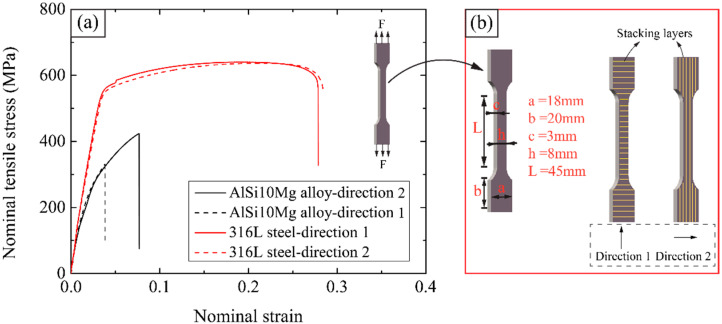
(**a**) The uniaxial tensile stress-strain relationship of SLM fabricated AlSi10Mg alloy and 316L steel parent material; (**b**) the geometric dimensions and SLM fabricating directions of tensile test specimen.

The uniaxial tensile tests of SLM manufactured AlSi10Mg alloy and 316L steel were performed based on GB/T 228.1-2010. The sketch of the dog-bone specimen is illustrated in Figure 3, where direction 1 and direction 2 represent the laser melting layers stacked along the loading direction and vertical to the loading direction, respectively. Dimensions of the dog-bone specimen are given in Figure 4b. The tensile response in the two-layer-stacking direction of the specimen was measured using a universal testing machine Instron^®^ 5581 with a load cell of 50 kN. The corresponding strain was recorded by an extensometer with a gauge length of 25 mm. The specimens were stretched at a constant loading rate of 2 mm/min to simulate a quasi-static loading condition. There were five repeats for each type of specimen test.

#### 2.3.2. In-Plane Compression Test of Hierarchical Honeycomb Specimens

In-plane compression tests were performed by the Instron^®^ 5581 universal testing machine, and the normal order honeycomb specimens were also tested for comparison. It should be noticed that the honeycombs were tested by applying load along the y direction in the present work. The load keeps a constant rate of 5 mm/min. The in-plane deformation changing and the failure derivation process were recorded by a low frame rate camera. The nominal compressive stress (*σ*) and strain (*ε*) can be calculated as *F*/*A* and *δ*/*L*, respectively, where *F* and *δ* indicate the in-plane transverse deformation and the corresponding force in the y-direction; *L* (= 53.6 mm) and *A* (= 46.4 × 25 mm^2^) represent the height along y-direction and the area of the cross-section in the x-z plane. Three parallel experiments were conducted for each type of honeycomb specimen.

## 3. Results and Discussion

### 3.1. Uniaxial Tensile Characteristics of SLM Manufactured AlSi10Mg Alloy and 316L Steel Material

Figure 4 demonstrates the stress-strain response of AlSi10Mg alloy and 316L steel along two SLM fabricating directions under a uniaxial tensile condition. The measured mechanical properties of each material have been summarized in Table 2, where the elastic modulus is calculated as the slope of stress-strain curves at initial elastic stage and the yield strength corresponds to a residual strain of 0.2%.

This indicates that the AlSi10Mg alloy fabricated along orthotropic directions exhibits significant anisotropy. When the deposited layers are stacked along the loading direction (i.e., direction 1), the material possesses lower elastic modulus, tensile strength as well as failure strain than that with layers stacked perpendicular to the loading direction (i.e., direction 2). There exists a significant anisotropy, which could stem from the asymmetric heat flux during laser irradiation and cooling [25] during the fabricate process layer by layer, leading to a large number of borderline pores in the specimen. The borderline pores make the specimen lack enough spaces for extensive deformation and are more likely to lead to specimen fracture. Therefore, a high stress level can be quickly reached along with the one-direction layer stacking because of the faster strengthening. The yield strength of the parent material is determined by 0.2% offset yield strength, which is defined as the amount of stress that will result in a plastic strain of 0.2%. The yield strengths in the two-layer stacking directions are identical, i.e., *σ_ys_* = 200 MPa. For 316L steel specimens, the anisotropy in terms of the mechanical properties in these two manufacturing directions is not obvious. Compared with AlSi10Mg alloy, the 316L steel exhibits 2.62 times and 6.45 times higher ductility in each direction. The effect of mechanical properties on in-plane compressive behavior will be discussed in the following sections.

### 3.2. In-Plane Compressive Behavior of Hierarchical Honeycombs

#### 3.2.1. Compressive Stress versus Strain Relations

**Figure 5:**
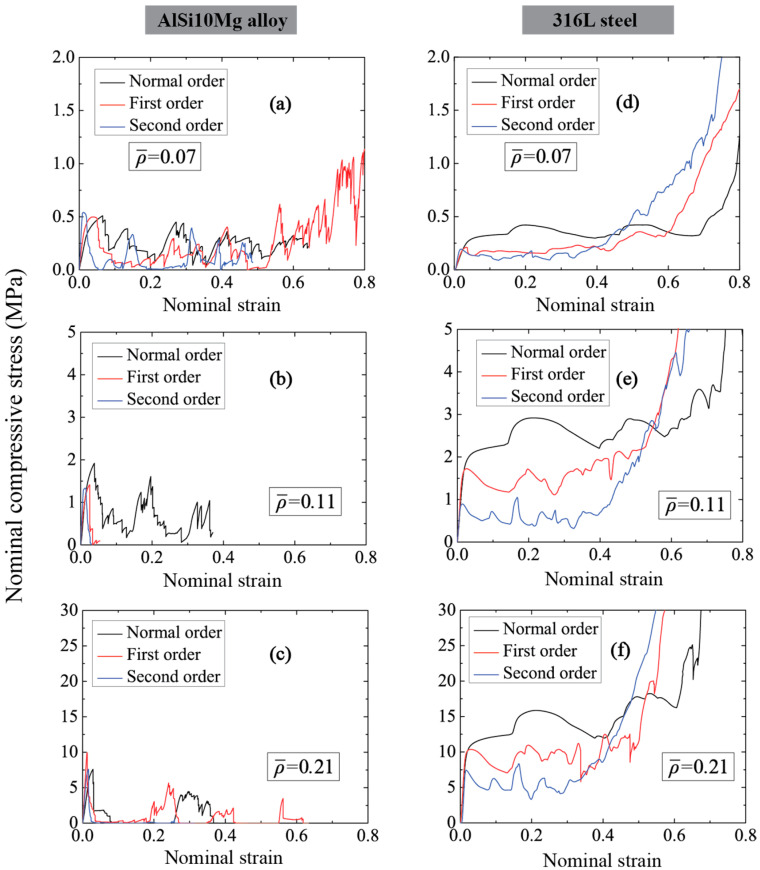
Compressive stress-displacement relationships considering different relative densities (**a**–**c**) AlSi10Mg alloy honeycombs (**d**–**f**) 316L steel honeycombs.

The nominal compressive stress versus strain relationships of three types of honeycomb made from AlSi10Mg alloy and 316L steel, respectively, have been plotted in Figure 5. Here, the compressive strength ($\sigma_{m}$) is defined as the peak stress before achieving decreased stress or crushing stress. As for AlSi10Mg alloy honeycombs (see Figure 5a–c), the normal order honeycombs exhibit strain-hardening behavior before achieving compressive strength, and this behavior is weakened in both hierarchical honeycombs which behave with nearly an elastic-brittle response under in-plane compression. All the AlSi10Mg alloy honeycombs experience catastrophic failure after compressive strength, and lower residual compressive stress is obtained by hierarchical honeycombs compared to normal order honeycombs. Unlike AlSi10Mg alloy honeycombs, there is no sharp stress decrease in 316L steel honeycombs and the in-plane compressive response can be divided into three stages: (i) linear elastic stage, (ii) crushing stress stage, and (iii) densification stage. With the increase of order of hierarchy, the honeycombs exhibit lower crushing stress and densification strain, whereas the densification stress of honeycombs is higher at large nominal strain.

#### 3.2.2. Deformation Modes of AlSi10Mg Alloy Hierarchical Honeycombs

**Figure 6:**
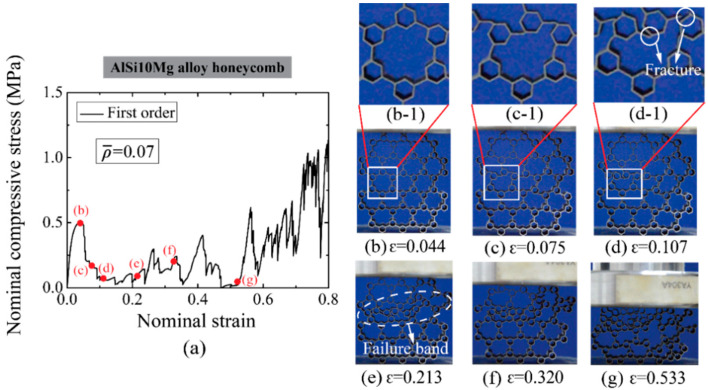
(**a**) The nominal compressive stress-strain relation of first-order AlSi10Mg alloy honeycomb with $\bar{\rho }=$ 0.07 and the deformation modes at typical strains are shown in (**b**–**g**). The white dashed line circle represents the fracture location of unit cells.

**Figure 7:**
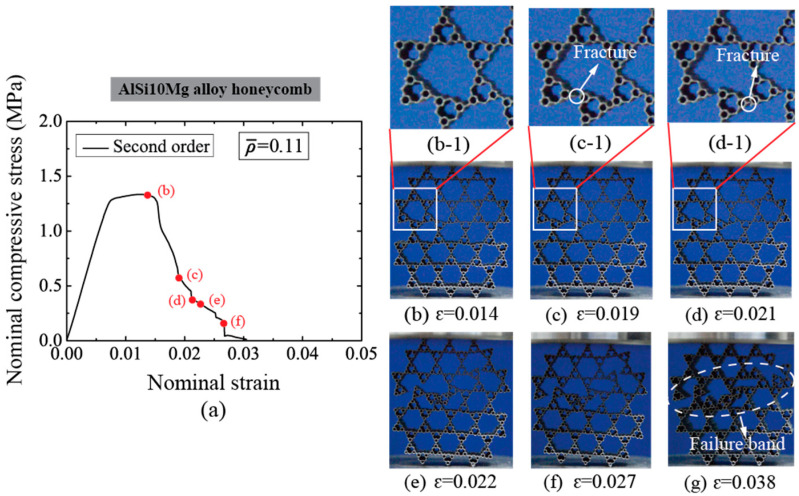
(**a**) The nominal compressive stress-strain relation of second-order AlSi10Mg alloy honeycomb with $\bar{\rho }=$ 0.11 and the deformation modes at typical strains are shown in (**b**–**g**). The white dash line circle represents the fracture location of unit cells.

The deforming process under an in-plane compression of selected AlSi10Mg alloy honeycombs at low relative densities are given in Figure 6 for the first-order honeycomb ($\bar{\rho }=$ 0.07) and Figure 7 for the second-order honeycomb ($\bar{\rho }=$ 0.11), respectively. It can be observed that both hierarchical honeycombs give rise to cell wall deformation at the joints of original and secondary unit cells before the compressive strength is achieved, which is the main reason leading to a distortion of original unit cells. However, the unit cells with a high order of hierarchy deform negligibly at initial compressive stage, owing to the higher bending resistance. Followed by the generation of fracture in the progressive wall and the collapse in the unit cell, a failure band develops in the hierarchical honeycombs, see Figure 6e and Figure 7g. The failure band diminishes the in-plane compression resistance considerably.

**Figure 8:**
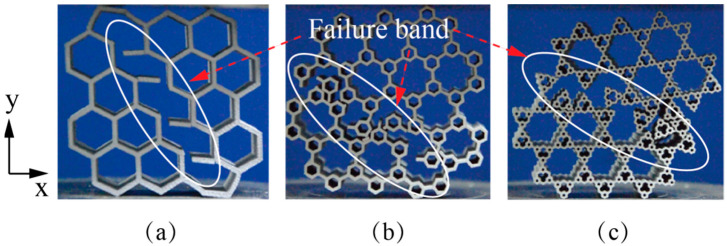
Failure modes and the failure bands of lSi10Mg alloy honeycombs ($\bar{\rho }=$ 0.21) (**a**) normal order, (**b**) first-order and (**c**) second-order.

Combining Figure 5c with Figure 8, it can be observed that there is no apparent cell wall bending phenomenon before failure and the failure band generated at the moment of achieving compressive strengths at a high relative density of $\bar{\rho }=$ 0.21. Compared with the low relative density honeycombs, the failure bands of high relative density honeycombs arise more catastrophically with wall joint fracturing along a specific inclined angle simultaneously. This could be attributed to the fact that the deformation of high relative density honeycombs is not dominated by the cell wall bending, which contributes to considerable elastic-brittle behavior under compression. In addition, the refined hierarchical order increases the inclined angles of failure bands with respect to the x-axis, showing as 122°, 139° and 155°, respectively.

#### 3.2.3. Deformation Modes of 316L Steel Hierarchical Honeycombs

**Figure 9:**
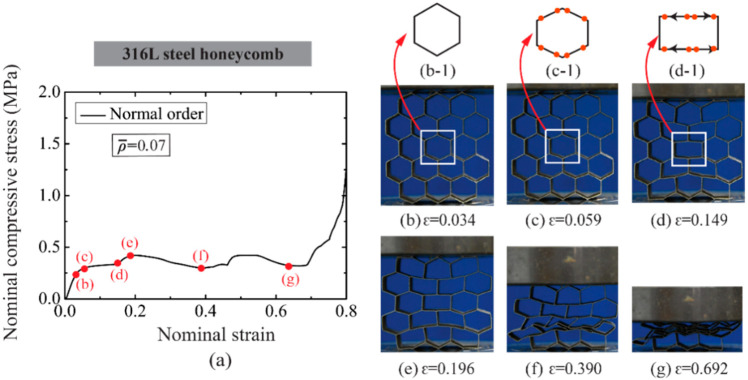
(**a**) The nominal compressive stress-strain relation of normal order 316L steel honeycomb with $\bar{\rho }=$ 0.07 and the deformation modes at typical strains are shown in (**b**–**g**). The red points in (**c-1**) and (**d-1**) represent the positions of plastic hinges.

**Figure 10:**
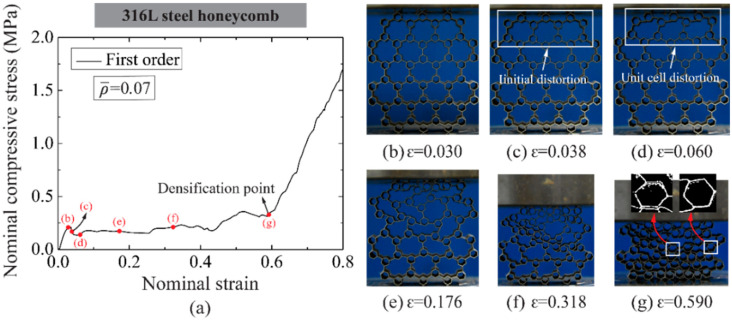
(**a**) The nominal compressive stress-strain relation of first-order 316L steel honeycomb with $\bar{\rho }=$ 0.07 and the deformation modes at typical strains are shown in (**b**–**g**).

**Figure 11:**
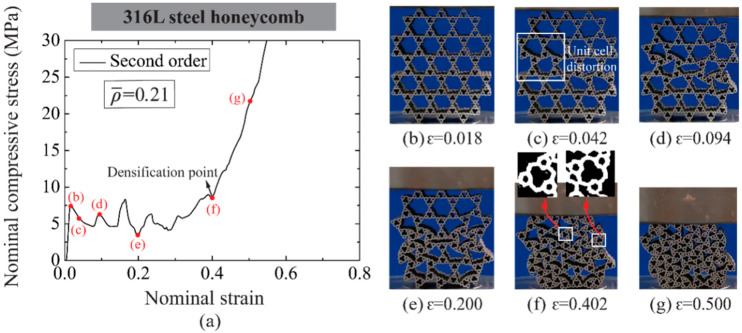
(**a**) The nominal compressive stress-strain relation of second-order 316L steel honeycomb with $\bar{\rho }=$ 0.21 and the deformation modes at typical strains are shown in (**b**–**g**).

Figure 9, Figure 10 and Figure 11 illustrate the deformation process of selected 316L steel alloy honeycombs at different relative densities. By contrast with the AlSi10Mg alloy honeycombs, the catastrophic failure and sharp decrease of stress do not occur to 316L steel honeycombs which behave with an elastic response, plastic crushing response and densification response in sequence. The difference is ascribed to the more excellent ductility of 316L steel material that avoids stress concentration and significant cell wall fracture. The normal order honeycomb exhibits plastic stress enhancement at two phases of Figure 9c–e, as pointed in Figure 9a, and the enhancement can be explained based on the deformation of a unit cell in the honeycomb. At the phase of Figure 9c,d, the enhancement is due to the bending moment of cell walls and strain hardening of parent material, and the unit cell deforms to an irregular hexagon, see Figure 9c-1; at the phase of Figure 9d–e, the further enhancement is owing to the bending moment as well as the tension of cell walls, and the unit cell deforms to be nearly rectangular at this moment, as sketched in Figure 9d-1. 

Take the first-order honeycomb of $\bar{\rho }=$ 0.07 and second-order honeycomb of $\bar{\rho }=$ 0.21 for example, as shown in Figure 10 and Figure 11, respectively, both hierarchical honeycombs exhibit similar stress–strain relations to normal order honeycombs under compression except for the strain softening after achieving compressive strength. The softening is detected along with original unit cell distortion (see Figure 10c and Figure 11c). Then the distortion develops progressively to a wider range of unit cells. Recall Section 3.2.1, the compressive strength and plastic crushing stress of hierarchical honeycombs are lower than those of normal order honeycombs at the same relative density. When secondary higher order unit cells deform, i.e., first-order unit cells for first-order honeycombs (see Figure 10g) and second-order honeycombs (see Figure 11f), the 316L steel hierarchical honeycombs start densification. Hence, it can be concluded that the in-plane compressive response is governed by the unit cells with distinct hierarchical orders, and the densification strain decreases with the refined order. In addition, it is interesting that both hierarchical honeycombs exhibit negative Poisson’s ratio behavior, especially for higher-order honeycombs, which is helpful to structural stability and energy absorption at large deformation [26]. 

### 3.3. In-Plane Failure Mechanism of Hierarchical Honeycombs

**Figure 12:**
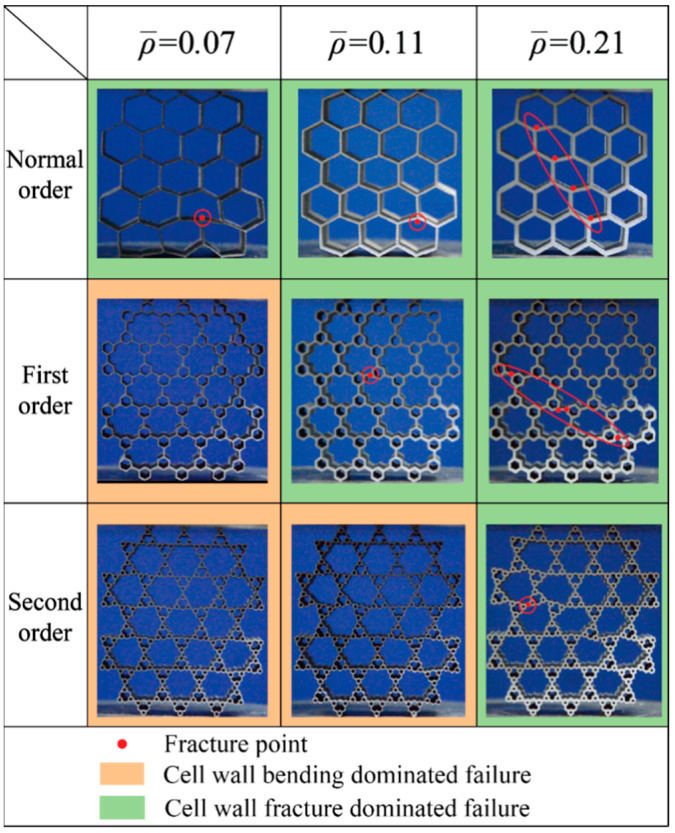
Failure modes when the compressive strengths are achieved.

Failure mode directly reflects the in-plane compressive behavior. Figure 12 demonstrates the failure modes of AlSi10Mg alloy honeycomb at the moment that the compressive strength is achieved. The color in this figure represents different failure modes of unit cells; the red marks represent the fracture positions of cell walls. It is shown that normal order honeycombs failed with wall fracture when the relative density ranges from 0.07 to 0.21. With the increase of hierarchical order, the failure mode of wall fracture is obtained at higher relative density, i.e., the first-order honeycombs fail due to a wall fracture at a relative density of 0.11 or 0.2; while the second-order honeycombs fail due to wall fracture only at a relative density of 0.21. Furthermore, all the wall fracture positions of hierarchical honeycombs are adjacent to the joints between original unit cells and secondary unit cells. At lower relative density, the first-order and second-order honeycombs fail with cell wall bending. However, there is no cell wall fracture observed in all the 316L steel hierarchical honeycombs when the compressive strength is achieved, and only cell wall bending and unit cell distortion can be observed. Hence, the failure mode photographs of 316L steel honeycombs are not presented for brevity.

In order to further identify the failure mechanisms of hierarchical honeycomb with different constituent materials, the first-order honeycomb is taken for example to be investigated. Haghpanah et al. [19] proposed five dominating failure mechanisms and theoretically analyzed the plastic collapse strengths of first-order honeycombs under an in-plane compression. According to the experimental observation, the bending failure mechanism (Mechanism I) matches the failure mode of the first-order honeycomb in the present study. As schematically shown in Figure 11a, the cell walls have bent before the honeycomb fails, and the critical positions are located at the wall joints between the original and the secondary higher-order unit cells. Based on Mechanism I [19], the theoretical in-plane compressive strength of the first-order honeycomb can be given as:

(2) $$\sigma_{m}=\frac{\sigma_{ys}}{4\left|1-\frac{\tan\theta }{\sqrt{3}}\right|{\left(1+2\gamma_{1}(2\eta_{1}-1)\right)}^{2}(0.5-\gamma_{1})}{\bar{\rho }}^{2}$$

where $\sigma_{ys}$ is the yield stress of constituent material; $\eta_{1}=1$. 

**Figure 13:**
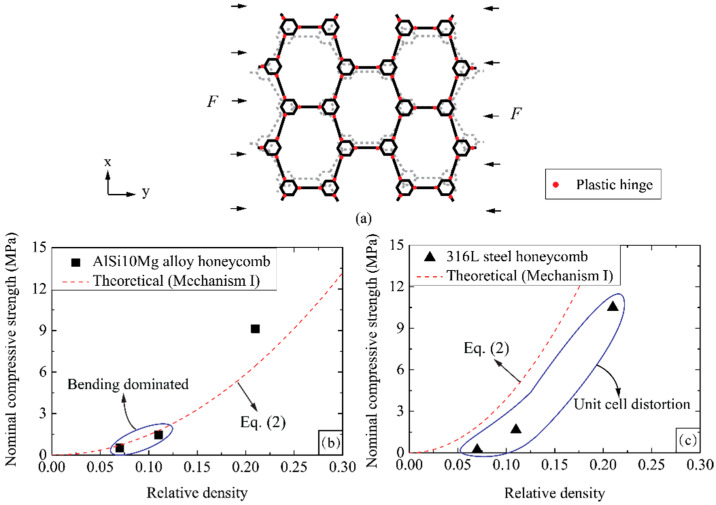
(**a**) The failure configuration of first-order honeycomb based on Mechanism I [19], Comparison between the in-plane experimental and theoretical predicted in-plane compressive strength in terms of (**b**) AlSi10Mg alloy and (**c**) 316L steel honeycombs as a function of relative density.

The experimentally measured and theoretically predicted compressive strength of first-order honeycombs are demonstrated in Figure 13b,c. Figure 13b shows that the theoretical predictions agree well with the experimental results at relative densities of $\bar{\rho }=$ 0.07 and 0.11, suggesting that the failure mode of AlSi10Mg alloy honeycomb is bending governed at low relative density. However, the discrepancy increases at a relative density of $\bar{\rho }=$ 0.21, thus the failure mode of AlSi10Mg alloy honeycomb is not only dominated by cell wall bending at high relative density. Gibson and Ashby [27] and Malek et al. [28] reported that the failure mode of honeycomb under in-plane compression is decided by the combination of bending, shearing and axial compression of cell walls. With the rising relative density, both shearing and axial compression of cell walls play a more and more important role during the failure process, leading to the transition of failure mode. Hence, due to the different contributions of three deformation modes to failure mechanisms, Equation (2) in terms of bending dominated failure is incapable of explaining the failure behavior of hierarchical honeycombs with high relative density.

Figure 13c shows that the testing compressive strengths of the 316L steel first-order honeycombs at different relative densities are normally lower than the theoretical predictions. Recall the mechanical properties of 316L steel in Section 3.1 and deformation modes of the first-order honeycomb in Section 3.2.3, it can be concluded that the parent material with significant ductility enhances the deformation capacity of unit cells, and the failure mechanism is governed by the unit cell distortion before cell wall bending. The contributions from axial compressive and shear deformation of cell walls are limited.

### 3.4. Comparison of Mechanical Properties between AlSi10Mg Alloy and 316L Steel Hierarchical Honeycombs

#### 3.4.1. Compressive Strength

**Figure 14:**
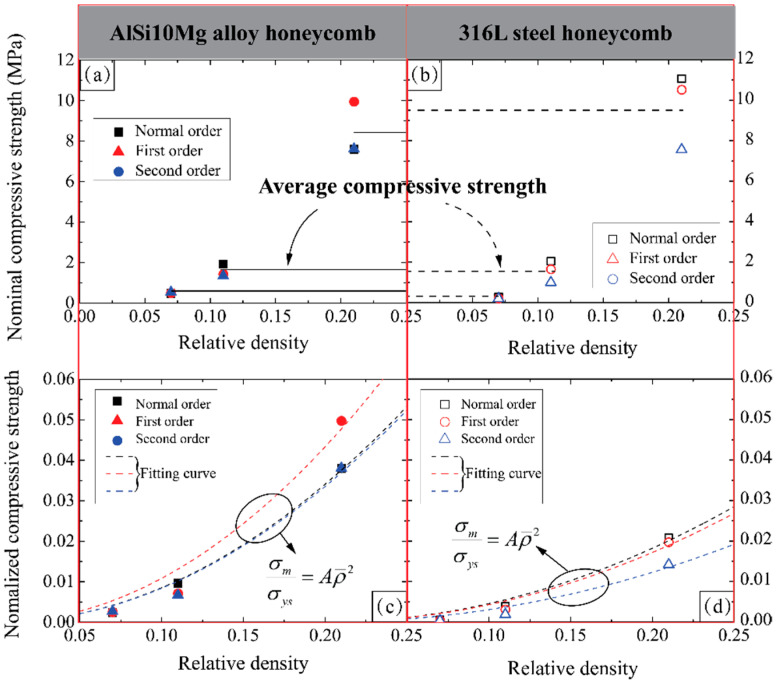
Nominal compressive strength of (**a**) AlSi10Mg alloy honeycombs and (**b**) 316L steel honeycombs, and the normalized compressive strength of (**c**) AlSi10Mg alloy honeycombs and (**d**) 316L steel honeycombs as a function of relative density. The fitting curve of scaling law for each type of honeycomb is plotted as well.

Table 3 summarizes the in-plane nominal compressive strength ($\sigma_{m}$) and elastic modulus (*E*) of three types of AlSi10Mg alloy and 316L steel honeycombs, and both the nominal and nominalized compressive strengths are plotted in Figure 14 for further investigation. According to Figure 14a, the AlSi10Mg alloy hierarchical honeycombs have similar compressive strengths to normal order honeycombs at same relative density. However, the compressive strength of 316L steel honeycombs decreases with the increase of hierarchical order at the same relative density, see Figure 14b, which may be due to the fact that higher-order honeycombs with thinner wall thickness are more likely to lead to structural instability and pre-distortion of unit cell. Although the 316L steel possesses considerably higher strength and elastic modulus than AlSi10Mg alloy, the compressive strengths of hierarchical honeycombs made from 316L steel are not significantly higher than those of honeycombs made from AlSi10Mg alloy, or even lower at low relative density, as the average level signed in Figure 14a,b. The explanation for this has been given in Section 3.3. 

To examine the effect of relative density, the compressive strengths are nominalized by the yield strength of their constituent material as shown in Figure 14c,d. The form of scaling law with respect to relative density is given as follows:

(3) $$\frac{\sigma_{m}}{\sigma_{ys}}=A{\bar{\rho }}^{2}$$

The values of the coefficient *A* for all the honeycombs have been summarized in Table 4. It is shown that the normalized compressive strength of honeycomb increases linearly with respect to the square of relative density, and the compressive strength of 316L steel a hierarchical honeycomb increases more slowly than that of an AlSi10Mg alloy hierarchical honeycomb.

#### 3.4.2. Elastic Modulus

**Figure 15:**
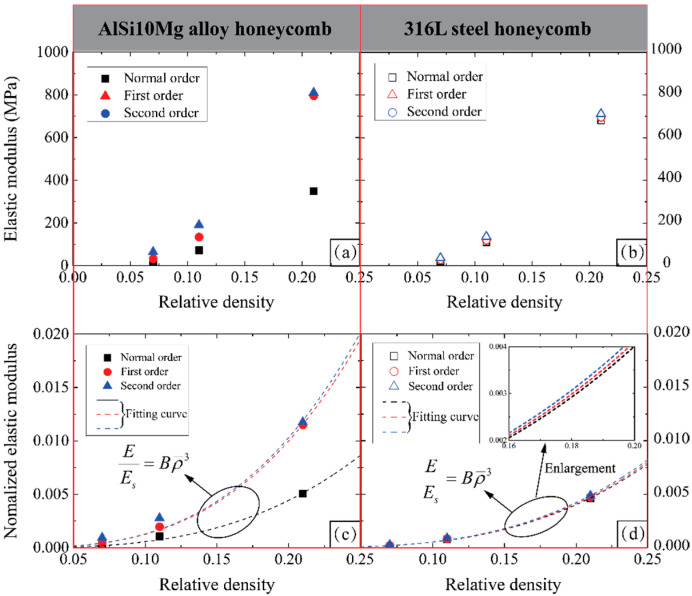
Elastic modulus of (**a**) AlSi10Mg alloy honeycombs and (**b**) 316L steel honeycombs; normalized elastic modulus of (**c**) AlSi10Mg alloy honeycombs and (**d**) 316L steel honeycombs vs relative density. The fitting curve of scaling law for each type of honeycomb is plotted as well.

Figure 15a,b show that, for AlSi10Mg alloy honeycombs, the hierarchical honeycombs possess higher elastic modulus than normal order honeycomb when the relative density is same, and could be further improved with the increase of hierarchical order. However, comparing with the normal order honeycomb, the hierarchical honeycombs made from 316L steel only possess slightly higher elastic modulus in terms of the same relative density. The distinction also stems from the mechanical properties of constituent material, which contributes to the different compressive elastic responses of hierarchical honeycombs. 

The compressive elastic modulus nominalized by the elastic modulus of their constituent material are shown in Figure 15c,d for examining the effect of relative density. A scaling law is given as below to show the relationship between the elastic modulus and the relative density [24,27]:

(4) $$\frac{E}{E_{s}}=B{\bar{\rho }}^{3}$$

The values of the coefficient *B* fitted are summarized in Table 5 for each type of honeycomb. The theoretical value of *B* for normal order honeycomb is calculated based on Gibson and Ashby [27]; The *B* value for hierarchical honeycombs are theoretically calculated according to Oftadeh et al. [24] based on Castigliano’s second theorem and an assumption of the Euler–Bernoulli beam. It indicates that the theoretical predictions for the coefficient of *B* are not consistent with the fitting values of experimental measurements. Moreover, the normalized elastic modulus of both the first-order and second-order 316L steel hierarchical honeycombs and the increasing trend with respect to the relative density are significantly lower than those of the AlSi10Mg alloy hierarchical honeycombs.

## 4. Conclusions

The characteristics of SLM additively fabricated self-similar hierarchical honeycombs with distinct relative density under in-plane compression have been experimentally identified. Also, both honeycombs made from AlSi10Mg alloy and 316L steel were tested aiming to explore the effect of the constituent material.

The hierarchical honeycombs made from AlSi10Mg alloy possess higher in-plane elastic modulus than normal order honeycomb, whereas they do not exhibit advantages in the compressive strength for the specific geometric parameters; the hierarchical honeycombs made from 316L steel exhibit lower compressive strength and there is only negligible elastic modulus enhancement with the increasing hierarchical order. All the failure positions of honeycombs were close to the interaction between original unit cell and secondary higher-order unit cell. The AlSi10Mg alloy honeycombs experience a failure mode transition from cell wall bending dominated to cell wall fracture dominated when the relative density and hierarchical order increase, while the 316L steel honeycombs fail with distortion of unit cells, followed by the cell wall bending. It is concluded that the failure mechanism depends on the wall thickness and parent material property, with different combinations leading to different contributions of cell wall bending, axial compression and shearing. Hence, the existing theoretical models based on Mechanism I can only predict the compressive strength of hierarchical honeycombs with low relative density and low ductile parent material. Although the mechanical behavior of AlSi10Mg alloy hierarchical honeycombs is superior to that of 316L steel hierarchical honeycombs before failure, the honeycombs with 316L steel exhibit negative Poisson’s ratio behavior that gives rise to more excellent structural stability and energy absorption capacity at large deformation. This is ascribed to the considerably higher ductility of 316L steel material. Moreover, the unit cells at different hierarchical orders deformed in sequence for 316L steel honeycombs and the densification was activated by the initial deformation of secondary higher-order unit cells.

## Figures and Tables

**Table 1:** In-plane geometric parameters of three types of honeycomb.

Materials	Numbering	t (mm)	$l_{0}\;(\mathrm{mm})$	$\gamma_{1}$	$\gamma_{2}$	$\bar{\rho }$
AlSi10Mg alloy/ 316L steel	AL-N7/ST-N7	0.40	6.7	-	-	0.069
	AL-F7/ST-F7	0.26		0.3	-	0.072
	AL-S7/ST-S7	0.2		0.3	0.12	0.080
	AL-N11/ST-N11	0.64		-	-	0.110
	AL-F11/ST-F11	0.42		0.3	-	0.116
	AL-S11/ST-S11	0.29		0.3	0.12	0.116
	AL-N21/ST-N21	1.28		-	-	0.220
	AL-F21/ST-F21	0.74		0.3	-	0.204
	AL-S21/ST-S21	0.54		0.3	0.12	0.216

**Table 2:** Tensile properties of SLM manufactured AlSi10Mg alloy and 316L steel.

Mechanical Properties		AlSi10Mg Alloy				316L Steel		
	Direction 1	Standard Deviation	Direction 2	Standard Deviation	Direction 1	Standard Deviation	Direction 2	Standard Deviation
Elastic modulus *E_s_*	71 GPa	3.5 GPa	53 GPa	3.2 GPa	147 GPa	9.7 GPa	147 GPa	10.3 GPa
Yield strength *σ_ys_*	200 MPa	5.8 MPa	200 MPa	13.4 MPa	533 MPa	21.4 MPa	524 MPa	24.3 MPa
Tensile strength *σ_t_*	423 MPa	11.2 MPa	331 MPa	20.9 MPa	640 MPa	29.7 MPa	639 MPa	37.8 MPa
Failure strain *ε_t_*	7.7%	0.67%	3.8%	0.29%	27.8%	1.81%	28.3%	1.94%

**Table 3:** Experimentally measured mechanical properties for AlSi10Mg alloy and 316L steel hierarchical honeycombs under in-plane compression.

$\bar{\rho }$	Honeycomb Type	AlSi10Mg Alloy Honeycomb		316L Steel Honeycomb	
		$\sigma_{m}\;(\mathrm{MPa})$	*E* (MPa)	$\sigma_{m}\;(\mathrm{MPa})$	*E* (MPa)
0.07	Normal order	0.47	17.75	0.28	20.09
	First-order	0.50	31.89	0.21	28.32
	Second-order	0.54	64.36	0.20	36.48
0.11	Normal order	1.92	72.37	2.05	110.45
	First-order	1.42	134.41	1.65	120.15
	Second-order	1.34	190.65	1.00	126.69
0.21	Normal order	7.59	349.49	11.06	680.80
	First-order	9.94	794.74	10.51	694.72
	Second-order	7.61	810.26	7.56	711.28

**Table 4:** Summary of the values of coefficient *A* for scaling law.

Honeycomb Order	AlSi10Mg Alloy	316L Steel
Normal	0.8516	0.4558
First	1.0826	0.4298
Second	0.8379	0.3072

**Table 5:** Summary of the values of coefficient B for scaling law.

Honeycomb Order	Theoretical Predictions [24,27]	Experimentally Fitted	
		AlSi10Mg Alloy	316L Steel
Normal	1.50	0.5528	0.5078
First	2.93	1.2464	0.5125
Second	5.07	1.2859	0.5169
